# Natural Isothiocyanates Block Adhesion and Invasion of Gemcitabine- and Cisplatin-Resistant Bladder Cancer Cell Lines

**DOI:** 10.3390/molecules31030555

**Published:** 2026-02-05

**Authors:** Jochen Rutz, Timothy Grein, Marina Laqua, Kenza Benhassine, Eren Perktas, Jindrich Cinatl, Anita Thomas, Felix K.-H. Chun, Axel Haferkamp, Eva Juengel, Igor Tsaur, Sascha D. Markowitsch, Roman A. Blaheta

**Affiliations:** 1 Department of Urology and Pediatric Urology, University Medical Center Mainz, 55131 Mainz, Germany; jochen.rutz@unimedizin-mainz.de (J.R.); timo.grein@hotmail.de (T.G.); anita.thomas@unimedizin-mainz.de (A.T.); axel.haferkamp@unimedizin-mainz.de (A.H.); emeweich@web.de (E.J.); igor.tsaur@med.uni-tuebingen.de (I.T.); sascha.markowitsch@unimedizin-mainz.de (S.D.M.); 2 Department of Urology, Goethe-University, 60590 Frankfurt am Main, Germany; marina.laqua@gmx.de (M.L.); s8244056@stud.uni-frankfurt.de (K.B.); erenperktas@outlook.de (E.P.); felix.chun@ukffm.de (F.K.-H.C.); 3 Institute of Medical Virology, Goethe-University, 60596 Frankfurt am Main, Germany; j.cinatl@kinderkrebsstiftung-frankfurt.de; 4 Department of Urology, University Hospital Tübingen, 72076 Tübingen, Germany

**Keywords:** natural isothiocyanates, bladder cancer, cisplatin resistance, gemcitabine resistance, adhesion, invasion

## Abstract

Aggressive metastatic progression often develops in bladder cancer patients with acquired cisplatin or gemcitabine resistance. The potential of the natural isothiocyanates allyl-isothiocyanate (AITC), butyl-isothiocyanate (BITC), and phenylethyl-isothiocyanate (PEITC) to inhibit adhesion and migration of cisplatin- or gemcitabine-resistant and sensitive RT112, T24, and TCCSUP bladder cancer cell lines was investigated. Parameters determined were: cell interaction with collagen or fibronectin, chemotaxis, and membrane receptors involved in adhesion (total and activated integrins β1, β4, β5, CD44s, and CD44v3-v7). CD44s’ location and adhesion- and migration-related signaling proteins were determined. AITC blocked adhesion of almost all sensitive and resistant cancer cells. PEITC and BITC suppressed fibronectin interaction of sensitive and resistant RT112. All three isothiocyanates diminished chemotaxis in all cell lines. Integrin expression was differentially altered but CD44s and CD44v were not altered. BITC and PEITC translocated CD44s from the cell membrane to cytoplasm. The tumor suppressor E-cadherin increased, whereas focal adhesion kinase (FAK), linked to integrin signaling, was deactivated after isothiocyanate treatment. Blocking FAK, β1, β4, or β5 was associated with reduced chemotaxis. Thus, AITC, BITC, and PEITC blocked adhesion and migration in cisplatin- and gemcitabine-resistant bladder cancer cells. This was associated with altered integrin expression and signaling, CD44s translocation, and enhanced E-cadherin.

## 1. Introduction

Based on GLOBOCAN 2022 cancer statistics, bladder cancer is the ninth most common cancer type worldwide. Over 610,000 new cases have been diagnosed with over 220,000 deaths in 2022 [1]. About 90% of all bladder cancer cases are related to urothelial carcinoma, which can further be divided into non-muscle invasive (NMIBC) and muscle invasive bladder cancer (MIBC). Treatment of MIBC is challenging and aside from surgery, cisplatin-based chemotherapy is generally administered. This consists of either MVAC (methotrexate, vinblastine, doxorubicin, cisplatin) or GC (gemcitabine, cisplatin). However, despite a high initial response rate, drug resistance rapidly develops, limiting the five-year survival rate to 39% for non-metastasized MIBC and less than 8% for metastasized MIBC [2]. Multimodal therapy, including immune checkpoint inhibitors, has significantly improved patient outcome [3]. Nevertheless, integration of atezolizumab into platinum-based chemotherapy only slightly elevates median overall survival by 2.6 months, compared to patients treated with chemotherapy alone [4]. The IMvigor210, Keynote 045 and 052 trials have demonstrated that the benefits of atezolizumab and pembrolizumab are limited to a subset of patients [5,6].

During the last years, cancer patients worldwide have become increasingly open-minded to complementary or alternative medicine (CAM) that is not part of conventional medical care. They include mind–body, manipulative, and biologically based therapies. The reasons to integrate CAM are manifold and include an active patient-controlled contribution to therapy, stimulating the immune system, improving quality of life, and reducing side effects of conventional treatment [7,8]. Of all CAM methods, herbal products are among the most frequently applied [9,10,11]. Indeed, integrating plant-derived compounds or extracts into conventional cancer treatment may improve patient outcome and treatment response. It may also increase quality of life and reduce negative side effects caused by radio- or chemotherapy. Several natural drugs have meanwhile become part of cancer management [12]. However, use is limited and their clinical relevance questioned, since evidence-based clinical trials are missing and phytochemical properties are not always thoroughly investigated.

The present investigation was designed to evaluate the potential of three natural isothiocyanates (ITCs), allyl-isothiocyanate (AITC), benzyl-isothiocyanate (BITC), and phenethyl-isothiocyanate (PEITC) on adhesion and migration properties of cisplatin- and gemcitabine-resistant bladder cancer cells in vitro. AITC, BITC, and PEITC are derived from plants of the Brassicaceae family. BITC originates from nasturtium, and AITC and PEITC are enriched in horseradish root. In vitro and in vivo experiments indicate distinct growth-blocking effects of the ITCs on several tumor entities [13]. However, the role of ITCs when resistance develops has not been clearly elucidated. We have recently shown that AITC, BITC, and PEITC suppress growth, proliferation, and clone formation of drug-resistant bladder cancer cell lines [14]. The present study indicates a significant influence of the compounds on invasive properties of bladder cancer cells resistant to cisplatin or gemcitabine. This is significant, since preventing metastatic tumor spread remains a major therapeutic hurdle.

## 2. Results

### 2.1. BITC, AITC, and PEITC Block Adhesion of Drug-Resistant Tumor Cells

**Figure 1:**
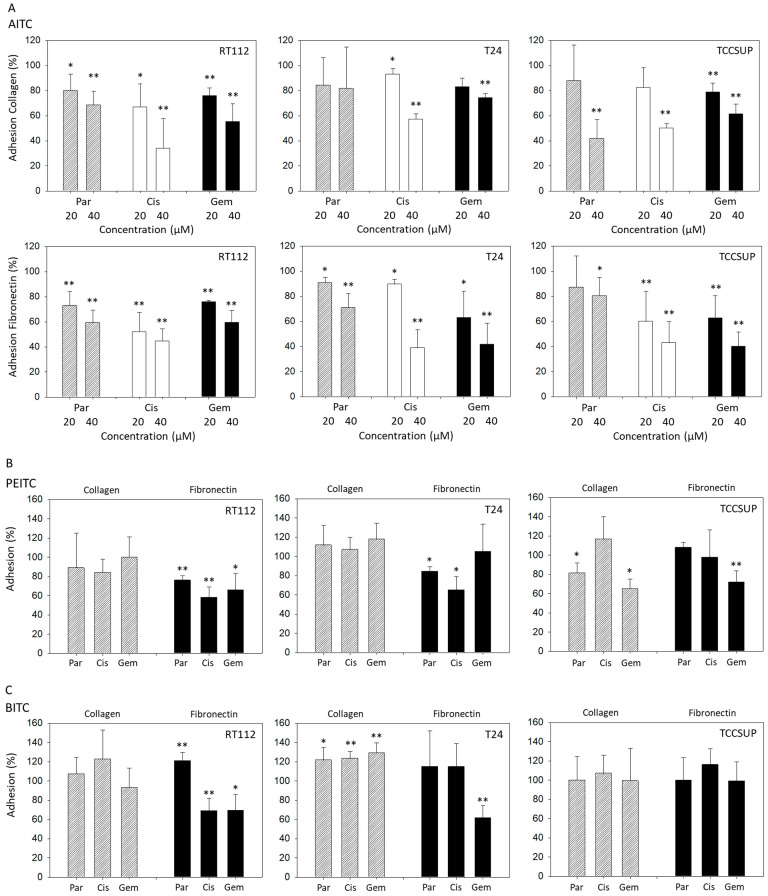
Adhesion of parental (Par), cisplatin- (Cis) and gemcitabine (Gem)-resistant RT112, T24, and TCCSUP bladder cancer cells to immobilized collagen or fibronectin following treatment with AITC (20 and 40 µM—panel (**A**)), PEITC (7.5 µM—panel (**B**)) or BITC (7.5 µM—panel (**C**)). Adhesion is given as mean relative to the respective untreated control (set to 100%). Error bars indicate standard deviation (SD), *n* = 3. * and ** indicate significant difference to the untreated controls with * *p* < 0.05 and ** *p* < 0.01.

Both 20 and 40 µM AITC significantly suppressed adhesion of parental, cisplatin- and gemcitabine-resistant RT112 cells to both collagen and fibronectin. Additionally, 20 and 40 µM AITC blocked adhesion of parental, cisplatin- and gemcitabine-resistant T24 cells to fibronectin, and adhesion of cisplatin-resistant T24 cells to collagen. However, AITC diminished the contact of gemcitabine-resistant T24 cells to collagen only at the higher dosage. AITC did not influence the binding of parental T24 to collagen. Similarly to the action in T24 cells, 20 and 40 µM AITC significantly reduced the attachment of cisplatin- and gemcitabine-resistant TCCSUP cells to fibronectin. Both concentrations also down-regulated binding of gemcitabine-resistant TCCSUP to collagen, whereas 40 µM (but not 20 µM) AITC effectively reduced binding of parental and cisplatin-resistant TCCSUP to collagen (Figure 1A).

PEITC blocked adhesion of all RT112 sublines to fibronectin but not to collagen. Similarly, T24 cell binding to collagen was not altered by PEITC, while binding of parental and cisplatin-resistant cells to fibronectin was blocked. Influence of PEITC on TCCSUP was different in that attachment of parental and gemcitabine-resistant cells to collagen, and attachment of gemcitabine-resistant cells to fibronectin, was significantly suppressed (Figure 1B).

Like PEITC, BITC diminished binding of RT112 cells (all sublines) to fibronectin but not to collagen. Binding of T24 cells (all sublines) to collagen was significantly elevated, compared to the untreated controls. Only T24 cells with established gemcitabine resistance lost contact with fibronectin. Exposing TCCSUP cells to BITC did not induce any changes in binding activity (Figure 1C).

### 2.2. Suppression of Chemotaxis and Motility

**Figure 2:**
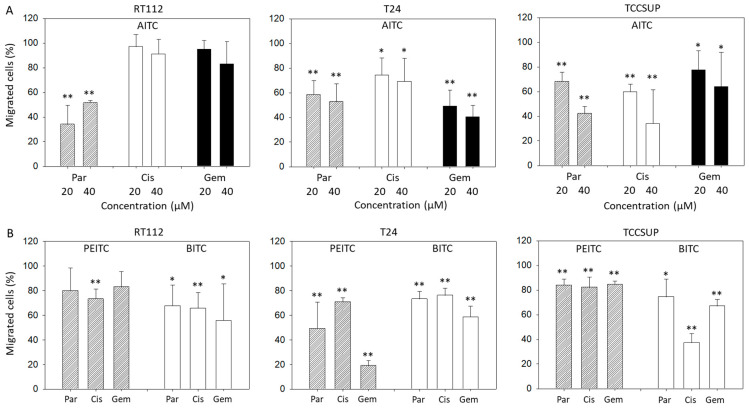
Influence of AITC (**A**), PEITC (**B**), and BITC (**B**) on chemotactic movement of parental (Par), cisplatin-resistant (Cis) or gemcitabine-resistant (Gem) RT112, T24, and TCCSUP cells. Values are given as percentage related to 100% controls (untreated cells). Error bars indicate standard deviation. Significant difference to the untreated controls: * = *p* < 0.05 and ** = *p* < 0.01. *n* = 3.

Chemotaxis of parental, cisplatin- and gemcitabine-resistant T24 and TCCSUP cells was distinctly blocked by 20 and 40 µM AITC. Both concentrations diminished chemotaxis of parental RT112 cells as well, whereas chemotaxis of cisplatin- and gemcitabine-resistant RT112 cells remained unchanged (Figure 2A). BITC treatment was associated with a significant loss of T24, RT112, and TCCSUP cells crawling underneath the Boyden chamber membrane. This effect was observed in all the parental, cisplatin- and gemcitabine-resistant cells. PEITC also down-regulated chemotactic movement of all cell lines and their respective sublines. However, diminished chemotaxis of parental (*p* = 0.09) and gemcitabine (*p* = 0.06)-resistant RT112 was not significant (Figure 2B).

**Figure 3:**
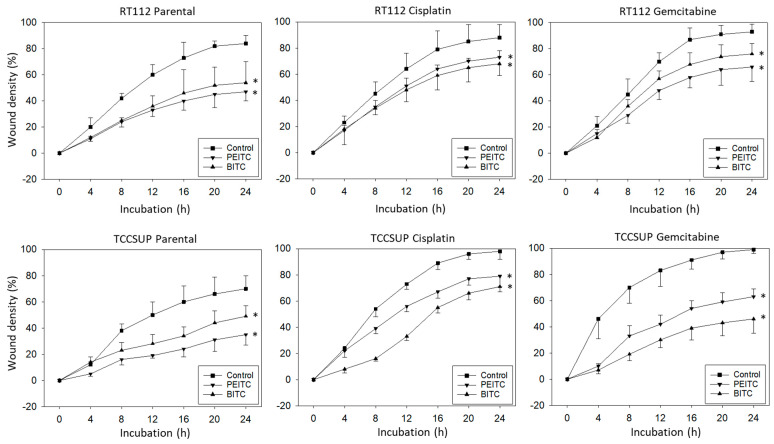
Influence of PEITC and BITC on wound closure from parental, cisplatin- and gemcitabine-resistant RT112 and TCCSup cells (compared to untreated controls). Values are shown as percentage related to 0% closure at zero time. * indicates significant difference to the untreated controls with *p* < 0.05 (*n* = 3).

Horizontal motility was evaluated in RT112 and TCCSUP cells treated either with PEITC or BITC. Both PEITC and BITC induced a significant reduction in motile activity of the parental and resistant cells in the wound-healing assay (Figure 3).

### 2.3. Alteration of Integrin Expression

**Figure 4:**
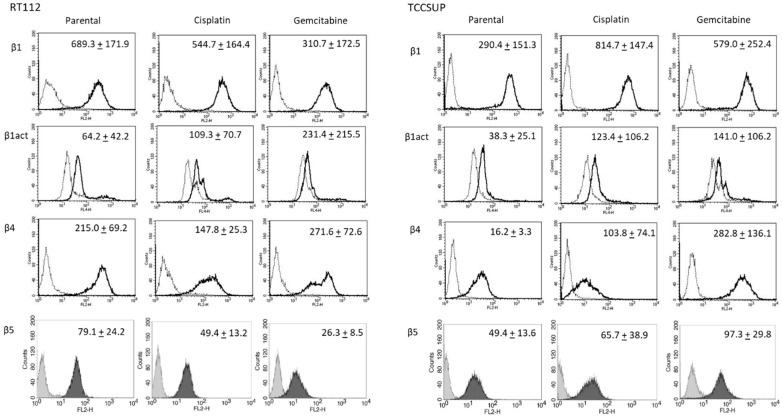
Integrin β subtype expression on parental, cisplatin- and gemcitabine-resistant RT112 and TCCSUP cells. Solid lines (β1, β1act, β4) and black filling (β5) show specific fluorescence; dashed line (β1, β1act, β4) and gray filling (β5) show isotype IgG1. The X-axis shows the relative fluorescence intensity (FL2-H) (given as relative logarithmic distribution), the Y-axis shows cell number (counts). Mean values +/−SD from *n* = 3 are shown in each set.

Further investigation was done with RT112 and TCCSUP cells, representing grade 2/3 (RT112) and grade 4 (TCCSUP) tumors. Figure 4 shows the integrin expression profiles of parental and resistant cell lines with MFU values (specific fluorescence). Higher levels of activated integrin β1 (phospho-integrin β1) were apparent in the cisplatin- and gemcitabine-resistant cells, compared to the parental cells. Integrin β4 was also highly expressed in cisplatin- and gemcitabine-resistant TCCSUP cells, compared to parental TCCSUP (Figure 4).

**Figure 5:**
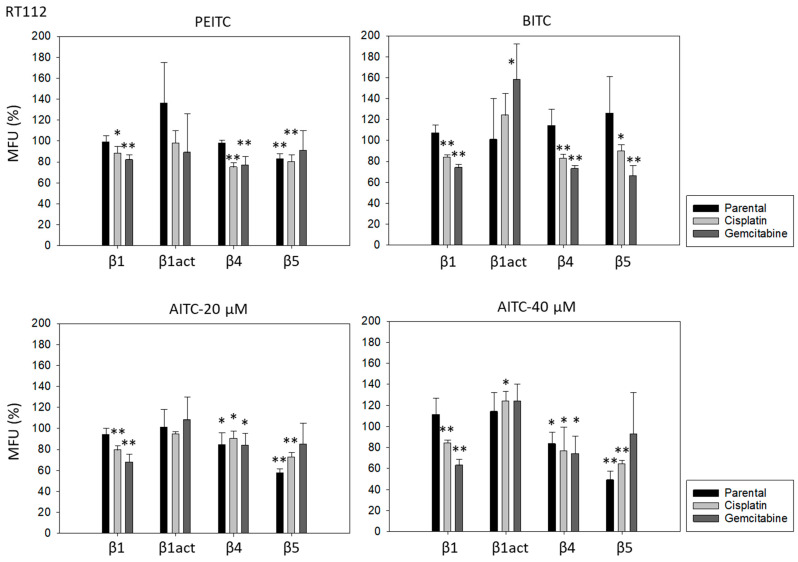

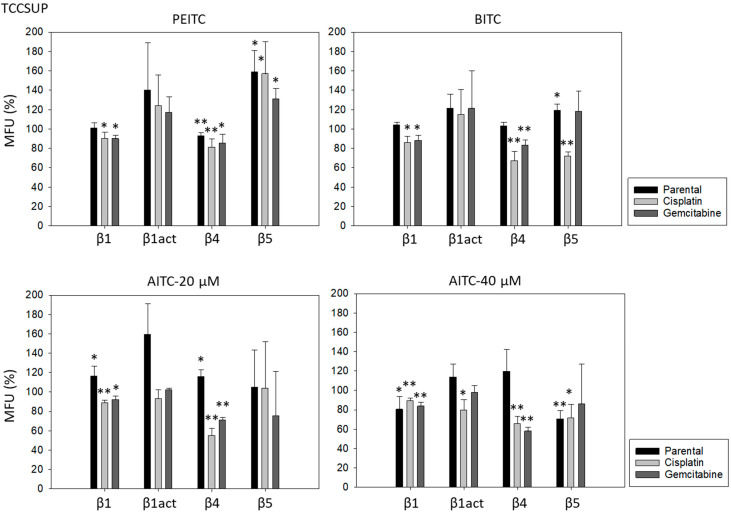
Integrin β1 (total and activated; act), β4, and β5 expression on parental, cisplatin- and gemcitabine-resistant RT112 und TCCSUP cells in the presence of PEITC, BITC, and AITC. MFU-values are given as means relative to the untreated controls (set to 100%). MFU: Mean Fluorescence Units. Error bars indicate SD. * indicates significant difference to untreated controls with *p* < 0.05. ** indicates significant difference to untreated controls with *p* < 0.01 (*n* = 3).

PEITC, BITC, and AITC diminished integrin β1 in both drug-resistant RT112 and TCCSUP cells (Figure 5). Additionally, 20 µM AITC slightly elevated integrin β1 in parental TCCSUP, but 40 µM AITC induced a reduction. Activated β1 was only altered by 40 µM AITC with an elevation in RT112 but a down-regulation in TCCSUP. All ITCs lowered integrin β4 in the drug-resistant cells (both RT112 and TCCSUP). Like integrin β1, β4 was slightly enhanced in parental TCCSUP by 20 µM AITC. Suppression of β4 was induced by 20 and 40 µM AITC in parental RT112. Integrin β5 was down-regulated in parental and cisplatin-resistant RT112 by PEITC and AITC, and down-regulated by BITC in cisplatin- and gemcitabine-resistant RT112. Different effects were seen in TCCSUP cells. PEITC evoked a significant increase in integrin β5 (all TCCSUP sublines). Integrin β5 was also elevated in the parental cells by BITC. Loss of this integrin type was only observed in cisplatin-resistant TCCSup by BITC and 40 µM AITC, and in parental TCCSUP by 40 µM AITC.

### 2.4. CD44 Expression and Translocation

**Figure 6:**
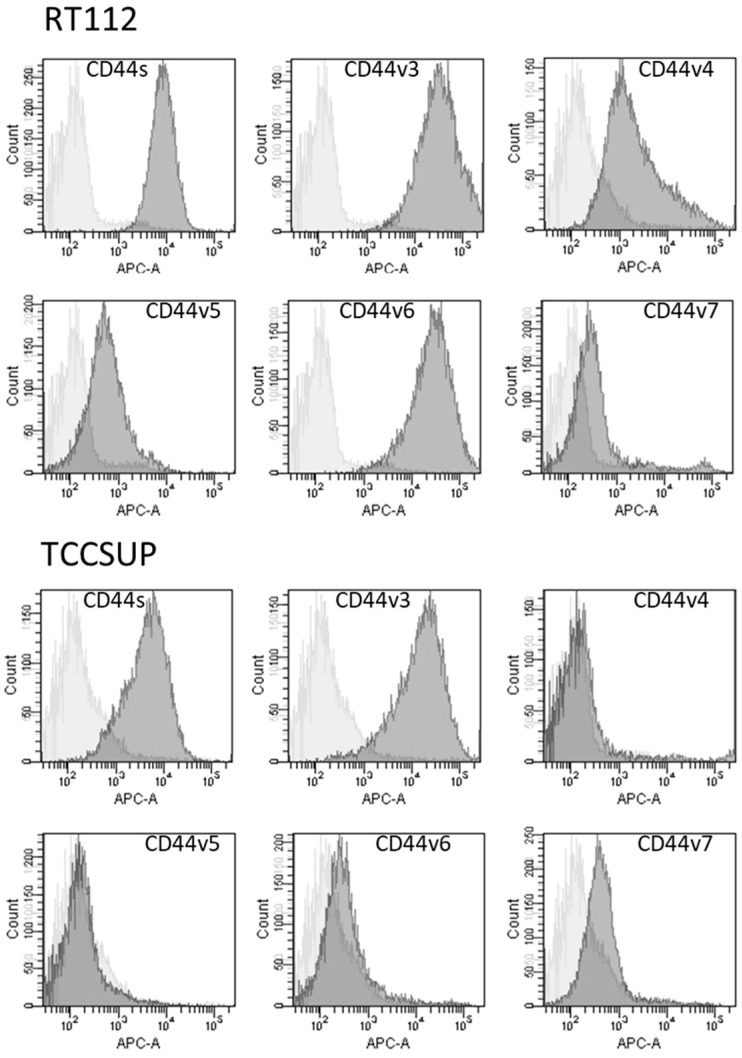
Surface expression of CD44 standard (CD44s) and CD44 variants v3, v4, v5, v6, and v7. The X-axis shows the relative fluorescence intensity (given as relative logarithmic distribution), the Y-axis shows cell number (counts). Dark gray filled areas show receptor-specific fluorescence, light gray filled areas show IgG1 isotype controls. One representative figure from *n* = 3.

CD44s and CD44v3 were strongly expressed on parental RT112 and TCCSUP cells. CD44v6 was also strongly expressed on RT112 but was only slightly detectable on TCCSUP cells. CD44v4, v5, and v7 were moderately present on both RT112 and TCCSUP as well with RT112 > TCCSUP (Figure 6).

**Figure 7:**
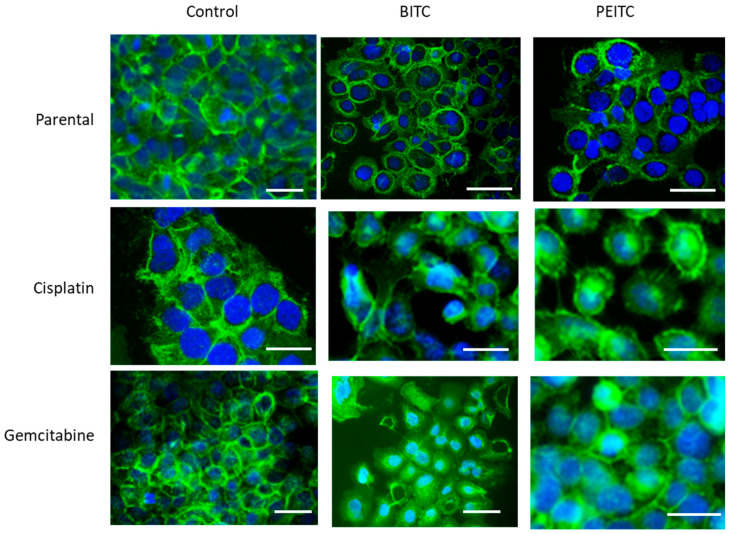
Distribution of CD44s in parental and drug-resistant RT112 after BITC or PEITC exposure. Pictures were taken with a Zeiss Axio Observer Z1, with a Plan-Neofluar × 40/1.3 oil immersion objective. Scale bar: 50 µm. Green color shows specific CD44s staining. Blue color shows counterstaining with DAPI.

No significant alterations in CD44s or CD44v expression were found in RT112 und TCCSUP (parental and resistant cells) following BITC, AITC, or PEITC exposure. However, CD44s receptor translocation was observed in RT112 cells by fluorescence microscopy. CD44s was particularly visible along the cell surface of the untreated control cells. Membranous accumulation was also observed when parental RT112 was treated with BITC or PEITC. However, CD44s was enriched in the cell cytoplasm following BITC or PEITC exposure to cisplatin- or gemcitabine-resistant RT112 (Figure 7).

### 2.5. Influence of BITC, AITC, and PEITC on Protein Expression

**Figure 8:**
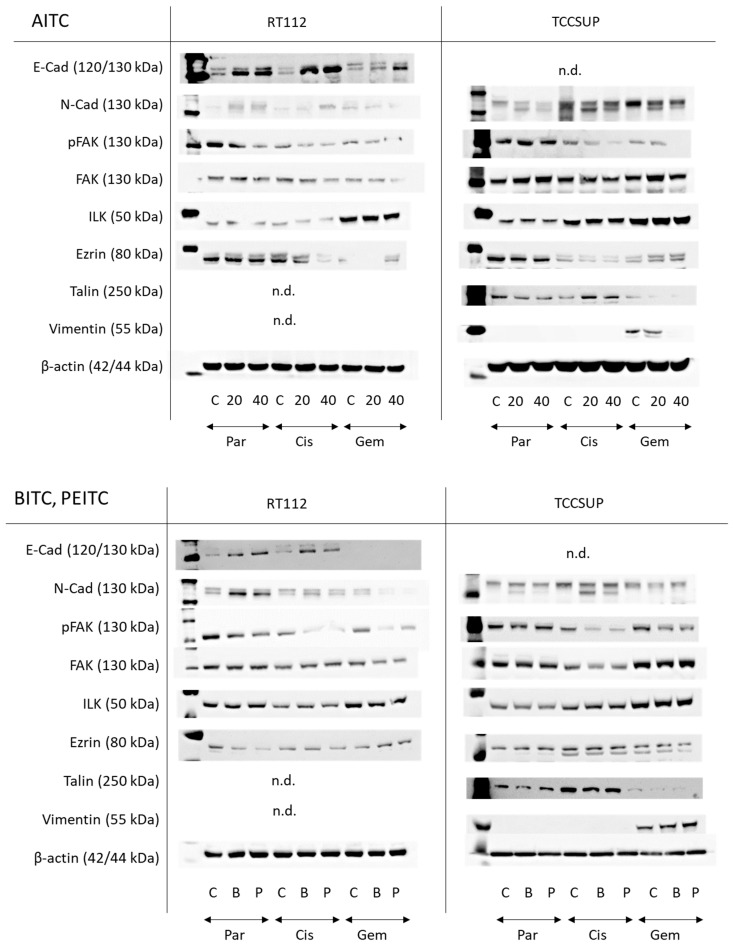
Western blots of adhesion- and migration-related proteins in parental (Par), cisplatin-resistant (Cis), and gemcitabine-resistant (Gem) RT112 and TCCSUP cells following AITC (20 and 40 µM), BITC (B; 7.5 µM) or PEITC (P; 7.5 µM) exposure for 24 h. β-actin was used to control protein loading and is representatively shown once. C indicates untreated controls. n.d. = non-detectable. E-cad = E-cadherin, N-cad = N-cadherin. All bands are representative of *n* = 3.

AITC markedly elevated E-cadherin in parental and drug-resistant RT122 cells. A very faint N-cadherin band appeared in untreated parental and drug-resistant RT112 cells. Expression was slightly elevated in parental and cisplatin-resistant cells but diminished in gemcitabine-resistant cells. pFAK was reduced in all resistant cell sublines by AITC, excluding TCCSUP controls where AITC did not alter pFAK expression. Loss of ezrin was also observed in cisplatin-resistant cells. Talin and vimentin were not visible. E-cadherin was not detectable in the TCCSUP cell line. N-cadherin, however, was strongly expressed, whereby AITC induced suppression. pFAK was reduced as well in the drug-resistant cells but not in the parental cells. Ezrin was diminished in the parental and cisplatin-resistant cells but elevated in cells resistant to gemcitabine. Talin was down-regulated in the parental and gemcitabine-resistant cells but up-regulated in the cisplatin-resistant cells. Vimentin was only detected in gemcitabine-resistant TCCSUP, whereby 40 µM AITC induced a complete loss of this protein (Figure 8 and Figure S1, Protein-Expression).

Both BITC and PEITC enhanced E-cadherin in parental and cisplatin-resistant RT112. N-cadherin was also elevated by both compounds in parental RT112, but diminished in the gemcitabine-resistant cells. Like AITC, pFAK was reduced in all resistant cell sublines by BITC and PEITC, and ezrin was diminished in the parental and cisplatin-resistant cells but elevated in gemcitabine-resistant RT112. N-cadherin was not distinctly modified by BITC or PEITC in TCCSUP. However, reduction in pFAK was seen in the drug-resistant cells following BITC or PEITC exposure. Vimentin was only expressed in gemcitabine-resistant TCCSUP (Figure 8 and Figure S1, Protein-Expression).

### 2.6. Blocking Studies

**Figure 9:**
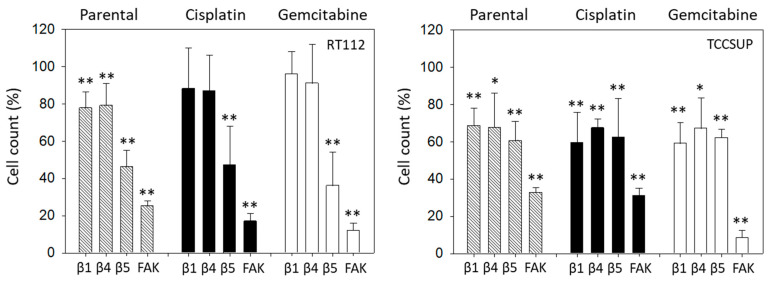
Influence of integrin β1, β4, β5 blockade or FAK suppression on the chemotactic movement of parental, cisplatin-resistant (Cisplatin), and gemcitabine-resistant (Gemcitabine) RT112 and TCCSUP cells. Cell number is expressed relative to unblocked controls (set to 100%). Error bars show standard deviation. Significant difference to related unblocked controls: * = *p* < 0.05; ** = *p* < 0.01; *n* = 3.

The relevance of integrins for migration in RT112 and TCCSUP was evaluated with the Boyden chamber assay. Blocking integrin ß1 or ß4 significantly suppressed chemotaxis of parental but not of cisplatin- or gemcitabine-resistant RT112 cells. However, blocking integrin ß5 and FAK considerably lowered chemotactic activity of all cell sublines (Figure 9, left). In contrast, blocking integrin ß1 or ß4 in TCCSUP cells diminished chemotactic activity of all cell sublines. The same was true with integrin ß5 function blocking antibodies and FAK blockade (Figure 9, right).

## 3. Discussion

The results show that the ITCs; AITC, BITC, and PEITC act on adhesion and invasion properties of both drug-sensitive and gemcitabine- and cisplatin-resistant bladder cancer cell lines. The drug concentrations employed in the present investigation (7.5 µM for BITC and PEITC, 20 and 40 µM for AITC) were based on a former study dealing with the influence of ITCs on bladder cancer growth and proliferation [14]. Similar concentrations have been used by other investigators, and 5 and 10 µM BITC reduced gastric and colorectal cancer growth by nearly 50% [15,16]. In another investigation, proliferation and invasion of mammary carcinoma cells were significantly blocked with 2.5–10 µM BITC [17]. Similarly, PEITC applied at 5–15 µM strongly suppressed growth and/or invasion of hepatocellular carcinoma [18,19], non-small cell lung cancer [20], and glioblastoma cells [21].

AITC was applied at higher concentrations, as was found necessary in previous investigations. It was shown that 20 and 40 µM AITC were necessary to reduce proliferation of human gastric cancer cells by 50% [22]. Additionally, 20 µM AITC has been shown to block human hepatoma cell proliferation by 50% [23], and the IC_50_ of AITC for oral squamous cell carcinoma cell growth reduction is approximately 25 μM [24]. Since PEITC and BITC were effective at lower concentrations than AITC, they may be superior to AITC in reducing adhesion, chemotaxis, and migration. Higher AITC concentrations have been found necessary to block proliferation of colon [25] and bladder cancer cells [14], compared to PEITC and BITC. BITC and PEITC have also shown stronger apoptosis-inducing effects than AITC in another bladder cancer cell model [26]. The reason for the different efficacy of AITC compared to BITC/PEITC is not clear. BITC and PEITC, but not AITC, clearly inhibited tubulin polymerization in cancer cells. Thus, BITC and PEITC functioned as anti-mitotic agents and the investigators assumed that the inferior effect of AITC on tubulin might be the reason why higher AITC concentrations were necessary to induce similar anti-proliferative activity [27]. Possibly, these differences in effective inhibitive concentrations might be due to the lipophilic and hydrophilic properties of the ITCs. BITC and PEITC are more lipophilic, while AITC is more hydrophilic [28]. The higher the lipid solubility, the higher the intracellular uptake. This would make BITC and PEITC more effective at lower concentrations than AITC.

Adhesion of tumor cells to the matrix proteins collagen and fibronectin was influenced differently by the ITCs. Adhesion of parental RT112 to collagen was reduced by AITC, and effectivity was not dependent on the concentration used. However, attachment of parental TCCSUP cells to collagen was only diminished at 40 µM AITC, while attachment of parental T24 cells was not altered at all by AITC. AITC profoundly blocked drug-resistant RT112 binding to collagen, whereas this was not the case when drug-resistant RT112 cells were treated with PEITC or BITC. Similar disparity has been reported in tumor cell–collagen interaction, where several bladder cancer cell lines responded differently to the ITC sulforaphane [29]. Since the bladder cancer cell lines used here reflect different grades of malignancy, the differentiation status of the tumor cells may at least partially be responsible for the degree of therapeutic response. Comparative analysis of RT112, T24, and TCCSUP cells revealed different expression of the epithelial–mesenchymal transition (EMT) markers E- and N-cadherin. Epithelial E-cadherin was detected in RT112 but not in T24 and TCCSUP cells. Mesenchymal N-cadherin instead was strongly expressed in cisplatin- and gemcitabine-resistant T24 and TCCSUP but not in resistant RT112 cells [29]. Treating RT112 cells with the ITC, sulforaphane, elevated E-cadherin expression in RT112 and was paralleled by a loss of tumor cell binding to fibronectin [29]. In good accordance, AITC up-regulated E-cadherin in RT112 cells in the present investigation and lowered the attachment of this cell line to collagen and fibronectin. E-cadherin serves as an essential component of adherens junctions and plays an integral part in cell adhesion. Up-regulation of E-cadherin has been shown to prevent adhesion of colon cancer cells to collagen and fibronectin, verifying the relevant role of this receptor in tumor–matrix interaction [30].

Nevertheless, concluding that E-/N-cadherin expression alone may define the response of bladder cancer cells to the ITCs may be oversimplified. In fact, BITC and PEITC did not alter RT112 adhesion to collagen and fibronectin, though E-cadherin was elevated. N-cadherin was even up-regulated by BITC and PEITC in sensitive RT112 cells. Treating a panel of bladder cancer cell lines, ordered into an epithelial-like cell cluster (among them RT112) and a mesenchymal-like cell cluster (among them T24 and TCCSUP) with a tyrosine kinase inhibitor, showed different effects on signaling pathways and cell function [31]. Therefore, the specific set of tumor proteins involved in differentiation processes may determine the response of different tumor cells to AITC, BITC, and PEITC. This is speculative and requires verification. In support of this speculation, growth- and proliferation-relevant pathways in T24 and RT112 cells were influenced by AITC, BITC, and PEITC differently [14], and the present investigation points to different expression of EMT-related proteins in TCCSUP compared to RT112 cells.

Differentiation dependent effects of the ITCs on the tumor cells were also seen in chemotaxis. All ITCs significantly suppressed chemotaxis of cisplatin- and gemcitabine-resistant T24 and TCCSUP cells, whereas AITC failed to do so with resistant RT112 cells. However, aside from cell-specific effects, compound-specific action should not be excluded. AITC, BITC, and PEITC are all characterized by an –N=C=S group, whereby the carbon atom interacts with cysteine thiols of cell proteins. However, aside from this similarity, the different side chains of AITC, BITC, and PEITC play relevant roles by influencing the electrophilicity of the –N=C=S group, leading to altered access to the carbon atom due to steric effects [32,33]. Different carbon activation might cause different effects on cell signaling. To what degree the side chains are involved in altering cell signaling pathways has not yet been evaluated.

In addition to investigating the influence of ITCs on chemotaxis, the horizontal migration under the influence of ITCs in TCCSUP and RT112 cells was also investigated. BITC and PEITC significantly blocked migration in the parental as well as the cisplatin- and gemcitabine-resistant sublines. These distinct anti-tumor effects in terms of adhesion and migration suggest that integrating AITC, BITC, and PEITC into a GC-based regimen may have therapeutic potential. In vitro and in vivo evidence has already shown that the ITCs overcome drug resistance to platinum-based chemotherapy in non-small cell lung [34] and cervical cancer cells [35]. Clinical trials on bladder cancer patients with established gemcitabine or cisplatin resistance have not yet been initiated. However, while screening for potentially therapeutic small-molecule drugs for patients with cisplatin-resistant lung squamous cell carcinoma, the ITC, sulforaphane, demonstrated potential in suppressing cisplatin resistance [36].

Since both integrin and CD44 are fundamentally involved in metastatic progression, in depth analysis of receptor expression was performed. BITC and AITC did not alter the CD44 expression level. However, they did induce intracellular enrichment of CD44s in gemcitabine- and cisplatin-resistant cells. The relevance of this can only be speculated upon. CD44 surface expression has been correlated with bladder cancer aggressiveness [37]. Correspondingly, lack of CD44s surface expression in tumor cells has been associated with a less invasive phenotype [38], and deletion of the intracellular CD44 domain has been shown to impair cell migration [39]. Therefore, translocation of CD44s into the intracellular space of resistant tumor cells as a result of BITC and AITC application could prevent the direct interaction of this receptor with the extracellular matrix. Intracellular CD44 fragments may interact with various cytoplasmic effectors which are also involved in regulating cell-trafficking [39]. Ongoing studies should, therefore, be directed towards investigating which proteins are affected in cells with established resistance against cisplatin and gemcitabine.

Blocking integrin β1 and β4 suppressed chemotaxis of the parental RT112 and TCCSUP cells, indicating a principal role of these receptors in tumor cell migration. Unexpectedly, β1 and β4 blockade down-regulated chemotaxis of the resistant TCCSUP but not of the resistant RT112 cells. The reason for this difference is not clear. The integrin β1 expression level distinctly differed among the cell lines and between sensitive and resistant cells. Integrin β1 was lower in resistant RT112 cells but elevated in resistant TCCSUP cells, each compared to their respective sensitive sublines. We assume that different basal integrin expression levels in both cell lines, with disparate alterations after resistance develops, activate adhesion- and invasion-related down-stream signaling differently. Gou et al. has shown that integrin β1 alterations correlate with chemotherapy resistance in a subset of urothelial carcinoma cells with enhanced EMT features [40]. This is important since integrins communicate with E–and N-cadherin [41,42]. We did not investigate EMT proteins in detail. However, drug-resistant TCCSUP cells were characterized by a loss of E-cadherin and high expression of N-cadherin. This contrasts with RT112 cells with high E-cadherin and low N-cadherin expression. Therefore, the different E- and N-cadherin expression levels during cadherin–integrin interaction may also explain why β1 blockade down-regulated chemotaxis of the resistant TCCSUP but not of the resistant RT112 cells. Resistance development has been associated with a functional switch of integrins so that integrin blocking may induce a different or even opposing response in sensitive versus resistant cells [43,44]. A functional switch of integrin β1 might contribute to the different role of this receptor in sensitive versus resistant RT112 cells as well. Although speculative, transcriptomic data of 262 gastric carcinoma samples has revealed an inverse correlation between E-cadherin and integrin β1 function [45], supporting our hypothesis.

Whether β4 acts similarly to β1 remains to be seen. Analysis of ovarian cancer TCGA datasets have revealed that integrin β4 might be involved in motile spreading when an initial low β4 level has been up-regulated in the course of cisplatin resistance [46]. In the present investigation, the β4 expression level was low in parental TCCSUP cells but high under cisplatin non-responsiveness. This could be relevant to β4 function. However, this is speculative and not supported by sound data. Independent from potential mechanistic and functional differences, the failure of integrin β1 and β4 antibodies to prevent chemotaxis of drug-resistant RT112 cells does not mean that the integrins are not relevant. Knocking down integrin β1 significantly inhibited proliferation and activated apoptosis of RT112 cells. This was associated with deactivation of the PI3K-Akt pathway [47]. There is evidence that integrin β1 promotes tumor growth by cross-communicating with the PI3K-Akt axis [48,49,50].

BITC, AITC, and PEITC decreased β1 and β4 in drug-resistant TCCSUP and RT112 cells. Whether this decrease correlates with Akt expression was not evaluated. However, BITC, AITC, and PEITC did reduce Akt in these cells in an earlier study, and the reduction was accompanied by diminished cell growth and proliferation [14]. The action of the ITCs is, therefore, highly relevant in regard to chemoresistance. Inhibition of integrin β1 has been demonstrated to enhance the sensitivity of lung, breast, and esophageal squamous cancer cells to cisplatin [51,52,53]. There is also evidence that targeting integrin β1 suppresses gemcitabine-resistant bladder cancer progression in vivo [40]. Integrin β4 overexpression attenuated cisplatin response of ovarian cancer cells [46], and knocking down integrin β4 improved cisplatin sensitivity in lung cancer [54]. Integrin β4 knockdown has also been shown to enhance gemcitabine’s effect on cholangiocarcinoma cells [55].

The integrin subtype β5 plays an essential role in the occurrence and development of various malignant tumors. High-level expression of β5 is associated with poor survival and advanced staging [56,57]. Inhibiting integrin β5 has been considered by other investigators to be an attractive therapeutic strategy when combined with cisplatin [58]. In good accordance, blocking integrin β5 stopped chemotactic movement of RT112 and TCCSUP cells, which makes β5 an interesting target in treating bladder cancer. However, AITC, BITC, and PEITC did not act similarly on the three tumor cell types. All the ITCs diminished integrin β5 in cisplatin-resistant RT112 but only BITC did so with gemcitabine-resistant cells. In contrast, integrin β5 was reduced in TCCSUP cells by BITC and 40 µM AITC, but integrin β5 in the gemcitabine-resistant cells was not diminished. PEITC rather up-regulated this receptor in TCCSUP. These results show different modes of action of the ITCs, and different pathways are influenced by them in RT112 and TCCSUP cells. Such inhomogeneous action is not uncommon. Treating a panel of head and neck squamous cell carcinoma cells with the integrin inhibitory peptide, cilengitide, β5 expression was diminished in several cell lines. However, β5 expression was not altered in one cell line, whereas β5 increased in another cell line [59]. Interestingly, growth and proliferation of the tumor cell line with a high β5 expression level was also reduced, opening questions about the precise action of this integrin.

BITC, AITC, and PEITC all diminished the expression of phosphorylated FAK in resistant TCCSUP and RT112 cells. Furthermore, FAK blockade by defactinib strongly inhibited chemotactic movement of these cells. FAK represents a key signaling molecule, which is not only connected to the integrins evaluated here but rather to a number of integrins that activate cellular pathways involved in cancer survival, proliferation, and migration [60]. Notably, high FAK expression has been correlated with drug resistance [61]. Due to its prominent role in oncogenesis, tumor progression and drug resistance, FAK targeting is considered a promising strategy to fight cancer and overcome drug induced resistance. Several FAK inhibitors have meanwhile been developed and subjected to clinical phase I and II trials [62]. Particularly, the combination of FAK inhibitors and chemotherapy has demonstrated clinical efficacy [63,64].

Based on the in vitro data presented here, we conclude that the ITCs may represent potent natural compounds worth integrating into a GC-based treatment regimen for bladder cancer. ITCs have been shown to resensitize other drug-resistant tumor cells. Long-term treatment with a panel of structurally diverse ITCs reversed the epithelial–mesenchymal phenotype and reduced the migratory potential of cisplatin-resistant lung cancer cells compared to parental cells. Reduced migration was accompanied with an increased expression of E-cadherin. Most importantly, the ITCs mediated a decrease in ATP Binding Cassette Subfamily C Member 1 (ABCC1) and aldehyde dehydrogenase (ALDH) 3 protein levels whose overexpression has been associated with drug resistance and cancer progression [65]. Similar effects have been noted with PEITC, which reduced the population of ALDH1-expressing cancer stem cells and suppressed the downstream multidrug resistance protein, P-glycoprotein (ABCB1) [66,67]. PEITC has also been shown to reverse cisplatin resistance in non-small cell lung cancer cells by depleting cellular glutathione (GSH). GSH-mediated export of cisplatin is a further key resistance mechanism with high expression correlated to a strong decrease in intracellular cisplatin, due to drug efflux [34]. PEITC has also been shown to restore chemosensitivity in cisplatin-resistant non-small cell lung cancer by targeting c-Myc and decreasing the Akt/mTOR signaling pathway [68]. This is important, since not only PEITC but BITC and AITC also decreased phosphorylated Akt in cisplatin- and gemcitabine-resistant RT112 and T24 bladder cancer cells [14]. Since AITC, BITC, and PEITC reverse chemoresistance in the bladder cancer cell model, bladder cancer patients may also profit from integrating ITCs into their treatment, once cisplatin and/or gemcitabine treatment is no longer effective.

## 4. Materials and Methods

Cell culture

RT112 and T24 cells were from ATCC/LGC Promochem GmbH, Wesel, Germany. TCCSUP cells came from DSMZ, Braunschweig, Germany. These cell lines were chosen because they represent different grades of bladder cancer. RT112 represents grade 2/3, T24 grade 3, and TCCSUP grade 4. All cells were cultured in Isocove’s Modified Dulbecco’s Medium (IMDM; Gibco/Invitrogen, Karlsruhe, Germany) supplemented with 10% fetal calf serum (FCS), 2% glutamax and 1% penicillin/streptomycin (all: Gibco/Invitrogen) in a humidified, 5% CO_2_ incubator. Subcultures from passages 7–24 were used, since culturing and passaging tumor cell lines over an extended period of time can cause undesired alterations of their genetic and molecular profiles [69].

Resistance was induced by exposing sensitive cells to either cisplatin or gemcitabine in increasing concentrations, up to a final dosage of 1 µg/mL (cisplatin—all cell lines) or 10 ng/mL (gemcitabine-TCCSUP), or 20 ng/mL (gemcitabine-T24, RT112) [29]. Resistance was verified as previously described [70]. Drug-associated toxic effects were determined by the trypan blue exclusion test (Gibco/Invitrogen). All investigations were based on comparing the behavior of drug-sensitive cells (termed parental cells) to that of drug-resistant cells.

AITC, BITC, and PEITC were from Merck, Darmstadt, Germany. Based on pilot studies [14], the tumor cells were treated with 7.5 µM BITC, 20 and 40 µM AITC, or 7.5 µM PEITC. Cell culture medium alone was used for controls.

Cell adhesion

To evaluate bladder cancer cell adhesion, 6-well multi-plates were either coated with collagen G type I (400 µg/mL dilution; Sigma-Aldrich, Taufkirchen, Germany) or with human plasma fibronectin (50 µg/mL dilution; BD Biosciences, Heidelberg, Germany). Uncoated plastic dishes served as controls. The multi-plates were then washed twice with bovine serum albumin (BSA, 1%) to prevent unspecific cell binding. Afterwards, 0.5 × 10^6^ parental or resistant cells (treated vs. non-treated) were added to each well for 60 min at 37 °C. Subsequently, the plates were again washed to remove cells not bound to the well bottoms. Adherent cells that had established firm bottom contact were then fixed with 1% glutaraldehyde (Sigma-Aldrich) and counted microscopically (×200 magnification) in five different fields (0.25 mm^2^) using a raster ocular. The mean number of adherent cells in the five fields was then calculated. Cell number was given in percentage related to respective controls set to 100%.

Chemotaxis

The Boyden chamber assay was used to investigate chemotactic movement towards a serum gradient. Parental and resistant tumor cells (0.5 × 10^6^ cells/mL) were pre-treated with either AITC, BITC, or PEITC for 60 min and then added to the upper chamber of a transwell system (Greiner Bio-One, Frickenhausen, Germany). The upper chamber was separated from the lower chamber by a membrane insert with 8 µm pores. To induce cell movement into the lower compartment, the upper chamber was filled with serum-free medium. The lower chamber contained medium enriched with 10% FCS as the chemoattractant. After 20 h incubation, cells that had not migrated underneath the membrane were removed from the upper membrane surface with a cotton swab. Cells migrating through the membrane and attached to the lower membrane surface were stained with hematoxylin and counted microscopically (×200 magnification). The mean number of migrated cells was determined from five different observation fields. The number of motile cells was given in percentage related to respective untreated controls set to 100%.

Scratch Wound Assay

The scratch wound assay was used to examine horizontal migration of TCCSUP and RT112 cells in the presence of the ITCs. Tumor cells were incubated with BITC or PEITC (37 °C, 5% CO_2_) for 4 h and then seeded onto 96-well ImageLock plates (Sartorius, Göttingen, Germany), previously coated with 400 µg/mL collagen G type I at 4 °C for 24 h (5 × 10^5^ cells/mL, 100 µL cell suspension per well). After reaching confluence, a defined scratch of about 700 µm was made with an IncuCyte^®^ WoundMaker (Sartorius). Detached cells were removed by washing with phosphate-buffered saline (PBS) containing Ca^2+^ and Mg^2+^. Cell culture medium without ITCs were applied to controls. Plates were then incubated in an IncuCyte^®^ Zoom (Sartorius) at 37 °C, 5% CO_2_ and photographed every 4 h for 24 h. Each experiment was done in triplicate. Relative wound density was calculated by the software WimScratch (Onimagin Technologies SCA, Córdoba, Spain).

Integrin expression

The bladder cancer cells were enzymatically detached from the bottom of the culture flasks with Accutase (PAA Laboratories GmbH, Pasching, Austria) and washed with 0.5% BSA (diluted in PBS; Gibco/Invitrogen). The cells were then incubated for 60 min at 4 °C with phycoerythrin (PE)-conjugated monoclonal antibodies targeting integrin β1 (anti-integrin β1 IgG1; clone MAR4, 20 µL), integrin β4 (anti-integrin β4 IgG2a, clone 439–9B (20 µL, all obtained from BD Biosciences, Heidelberg, Germany). Anti-integrin β5 (IgG2a; clone REA718, 2 µL) was from Miltenyi Biotech, Bergisch Gladbach, Germany. Anti-phospho-integrin β1 (Thr788/789, 2.5 µL; Merck KGaA, Darmstadt, Germany) was labeled with allophycocyanin (APC). Integrin expression on the cell surface was detected by a FACSCalibur flow cytometer (FL2-H (log) or FL4-H (log) channel histogram analysis, CellQuest Pro 4.0.2. software; both from BD Biosciences). Per scan 1 × 10^4^ cells were evaluated. Integrin expression was shown as mean fluorescence units (MFU). Unspecific fluorescence was evaluated by staining the cells with IgG1-PE (MOPC-21), IgG2b-PE (R35-38) or IgG2b-APC (all from BD Biosciences).

CD44 expression

Bladder cancer cells were removed from their culture flasks and washed with 0.5% BSA as previously described. Tumor cells were then stained with anti-CD44s (clone SFF-2, 2.5 µL), anti-CD44v3 (clone VFF-327v3, 2.5 µL), anti-CD44v4 (clone VFF-11, 5 µL), anti-CD44v5 (clone VFF-8, 25 µL), anti-CD44v6 (clone VFF-7, 2.5 µL), and anti-CD44v7 (clone VFF-9, 2.5 µL); all from eBioscience, ThermoFisher, Darmstadt, Germany). The antibodies had previously been conjugated with the Lightning-Link Allophycocyanin (APC) Conjugation Kit (eBioscience, ThermoFisher). APC (mouse IgG1 K, clone P3.6.2.8.1 from ThermoFisher, Dreieich, Germany) served as the isotype control. Receptor expression was analyzed by a FACSCalibur flow cytometer (FL4-H (log) channel histogram analysis, 1 × 10^4^ cells per scan) with CellQuest Pro 4.0.2 software (all from BD Biosciences) and expressed as MFU.

Localization of CD44s was additionally done with a fluorescence microscope (Zeiss Axio Observer Z1; Carl Zeiss AG, Jena, Germany). Parental and drug-sensitive RT112 cells (treated versus non-treated) were transferred to 8-well polystyrene culture slides (Falcon^®^; Merck Millipore, Darmstadt, Germany) and incubated at 37 °C, 5% CO_2_ for 72 h. Following incubation, cells were washed with PBS and fixed in cold (−20 °C) methanol/acetone (60/40 *v*/*v*). To prevent unspecific antibody binding, cells were then washed with BSA (0.5%). RT112 cells were subsequently incubated with fluorescein isothiocyanate (FITC) labeled CD44s (60 min, RT). To prevent photobleaching of the fluorescent dye, cells were embedded in an antifade reagent/mounting medium mixture including 4’,6-diamidino-2-phenylindole (DAPI; VECTASHIELD, Antifade Mounting Media, Biozol, Eching, Germany). Counter staining was done with 4’,6-diamidino-2-phenylindole (DAPI). Cells were viewed by fluorescence microscopy.

Western Blot Analysis

Lysates of TCCSUP and RT112 cells were applied to a 7–12% polyacrylamide gel (depending on the protein size) and electrophoresed for 90 min at 100 V. The protein was then transferred to nitrocellulose membranes (1 h, 100 V). After blocking with nonfat dry milk for 1 h, the membranes were incubated overnight with antibodies directed against the following targets: FAK (clone 77, dilution 1:1000), phospho-specific FAK (pFAK, pY397), clone 18, dilution 1:1000), ILK (clone 3, dilution 1:1000; all from BD Biosciences), Ezrin (polyclonal, dilution 1:1000), E-cadherin (clone 24E10, dilution 1:1000), N-cadherin (clone 13A9, dilution 1:1000), vimentin (clone D21H3, dilution 1:1000), and talin (clone C45F1, dilution 1:1000; all from Cell Signaling, Leiden, The Netherlands). HRP-conjugated goat anti-mouse IgG and HRP-conjugated goat anti-rabbit IgG (dilution 1:5000; both from Cell Signaling) served as secondary antibodies. The membranes were briefly incubated with ECL detection reagent (ECL; Amersham/GE Healthcare, München, Germany) to visualize the proteins and then analyzed by the Fusion FX7 system (Peqlab, Erlangen, Germany). β-Actin (clone AC-15 dilution 1:1000, Cell Signaling) served as the internal control.

Blocking studies

To investigate the relevance of the integrins in modulating chemotaxis, RT112 and TCCSUP cells (parental, cisplatin- and gemcitabine-resistant) were incubated with the function-blocking antibodies anti-integrin β1, anti-integrin β4, or anti-integrin β5. To block integrin-related signaling, the FAK-inhibitor defactinib (Biomol, Hamburg, Germany) was applied. Controls were not blocked. Subsequently, chemotaxis was carried out as described above.

Statistics

All experiments were performed three times. Statistical significance was calculated using ANOVA for adhesion and migration, and *t*-test for integrin expression and functional blockade. Differences were considered statistically significant at *p* < 0.05.

## 5. Conclusions

Outlook: Evidence has been provided that ITCs may diminish the risk of bladder cancer progression. The in vitro data presented here point to distinct tumor-suppressive properties of AITC, BITC, and PEITC in terms of adhesion and invasion blockade. Due to their natural origin, AITC, BITC, and PEITC are considered non-toxic and undesired side effects; should they occur, they would probably be moderate in nature. Therefore, combining these ITCs with conventional chemotherapy might make current treatment protocols more effective. Further investigation should be directed towards exploring whether ITC application can hinder drug resistance or even resensitize bladder cancer to cisplatin and/or gemcitabine.

## Data Availability

The original contributions presented in this study are included in the article/Supplementary Material. Further inquiries can be directed to the corresponding author.

## Supplementary Materials

The following supporting information can be downloaded at https://www.mdpi.com/article/10.3390/molecules31030555/s1, Figure S1: Protein Expression.
