# Supplementary material for: Natural Isothiocyanates Block Adhesion and Invasion of Gemcitabine- and Cisplatin-Resistant Bladder Cancer Cell Lines
Source: Molecules. 2026 Feb 5;31(3):555. doi: 10.3390/molecules31030555 (PMC12899539; doi:10.3390/molecules31030555)

Supplement S1, Protein-Expression, RT112 cells, BITC, PEITC

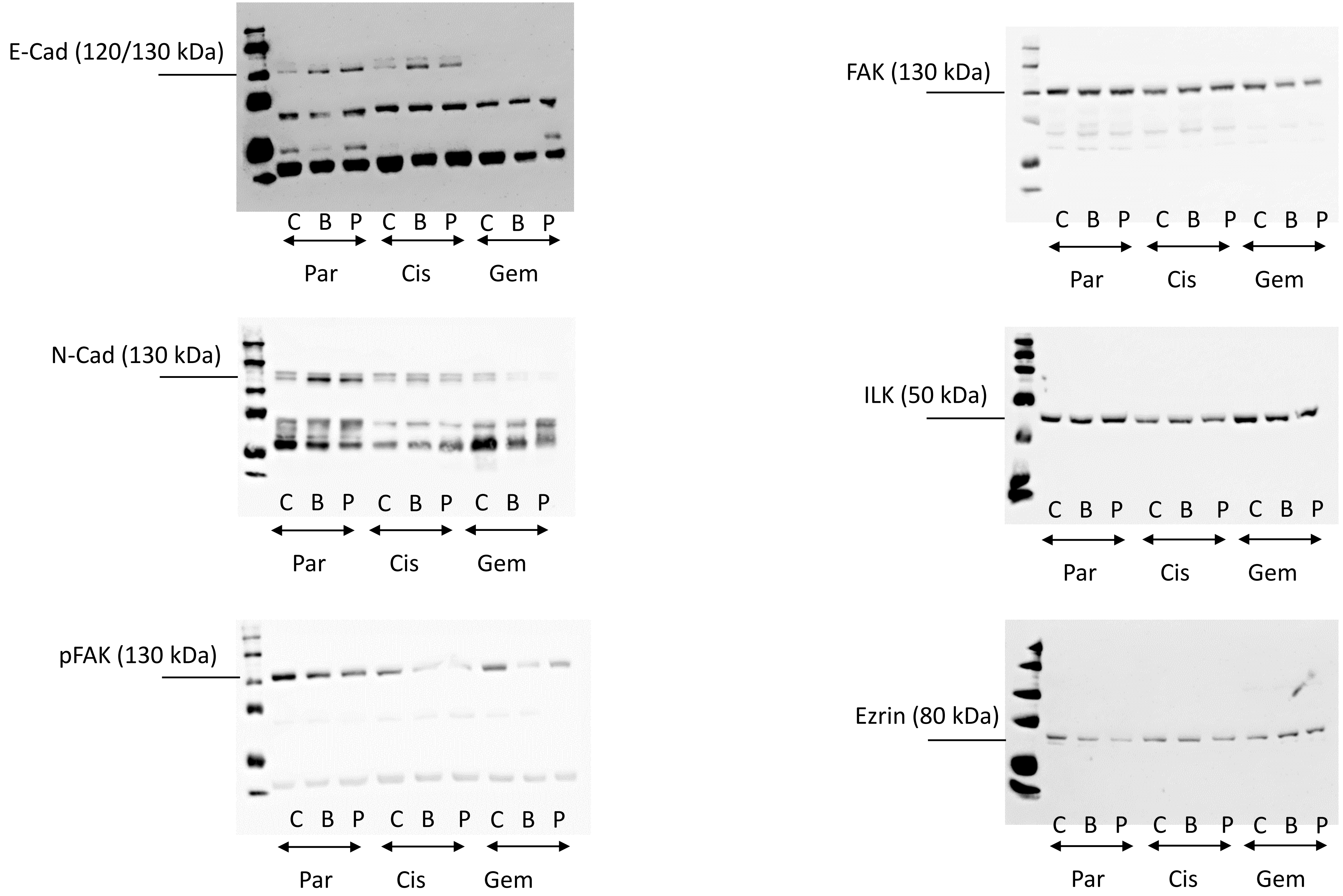

TCCSup cells, BITC, PEITC

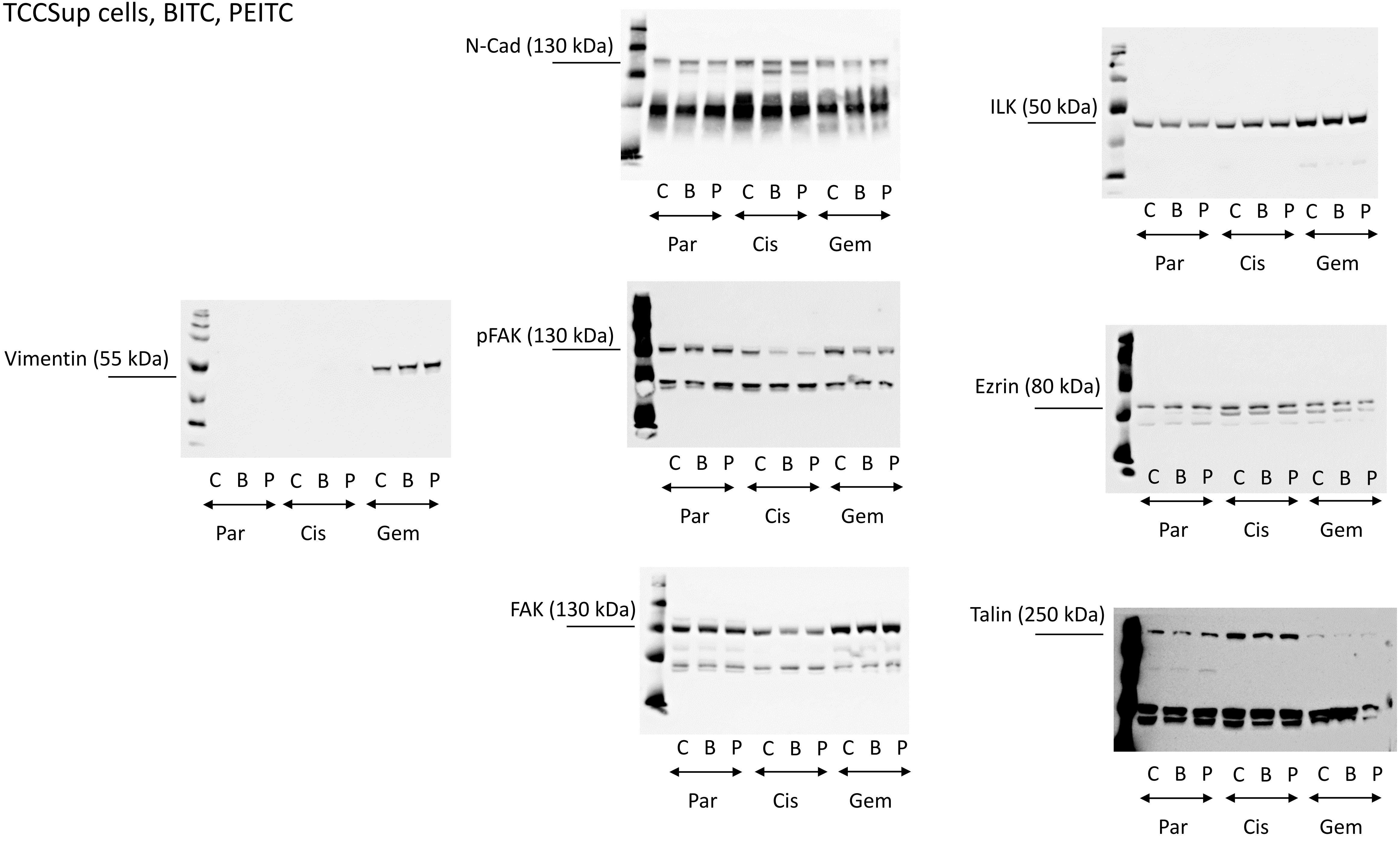

RT112 cells, AITC (Control C, 20 µM, 40 µM)

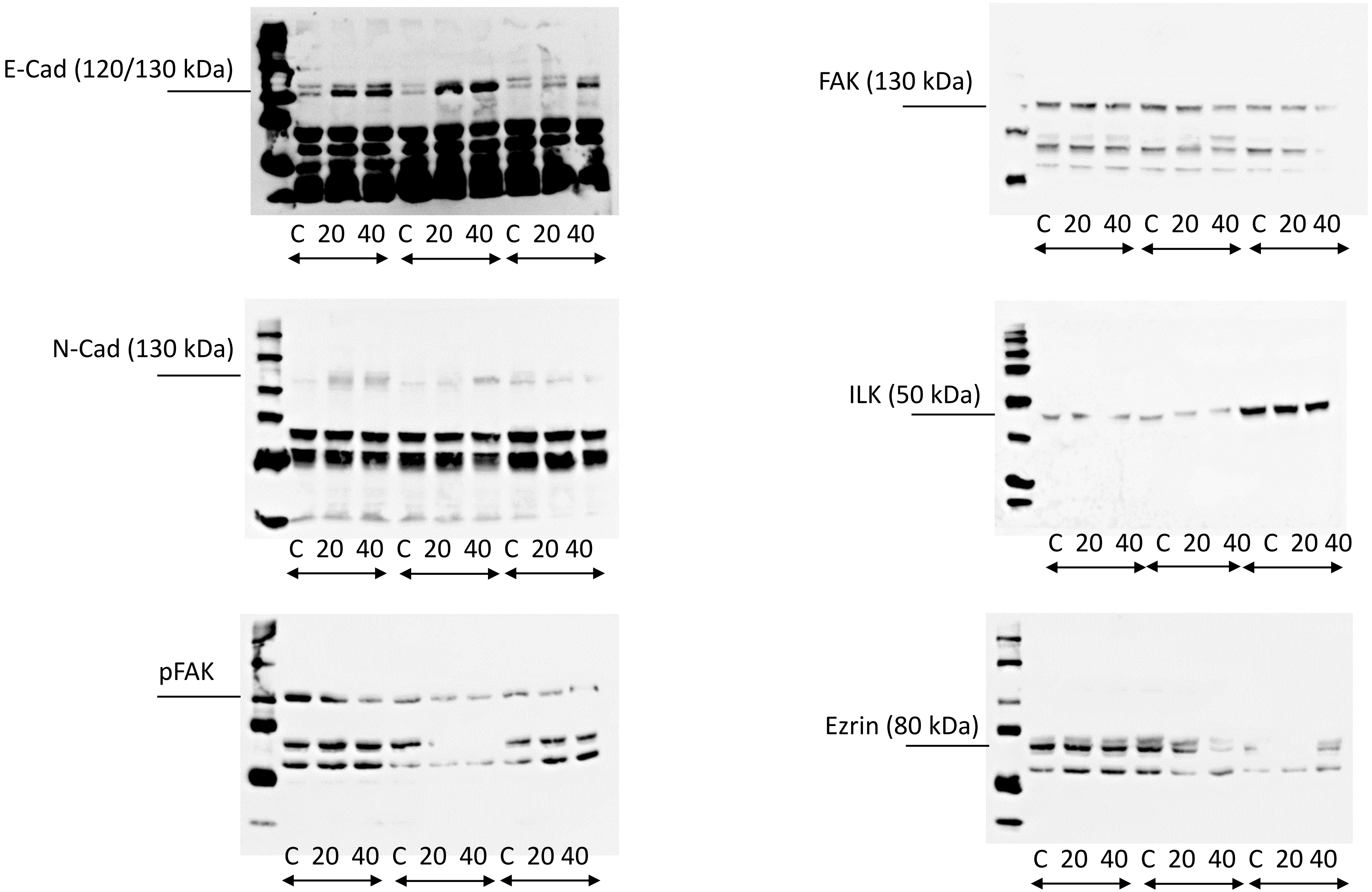

TCCSup cells, AITC (Control C, 20 µM, 40 µM)

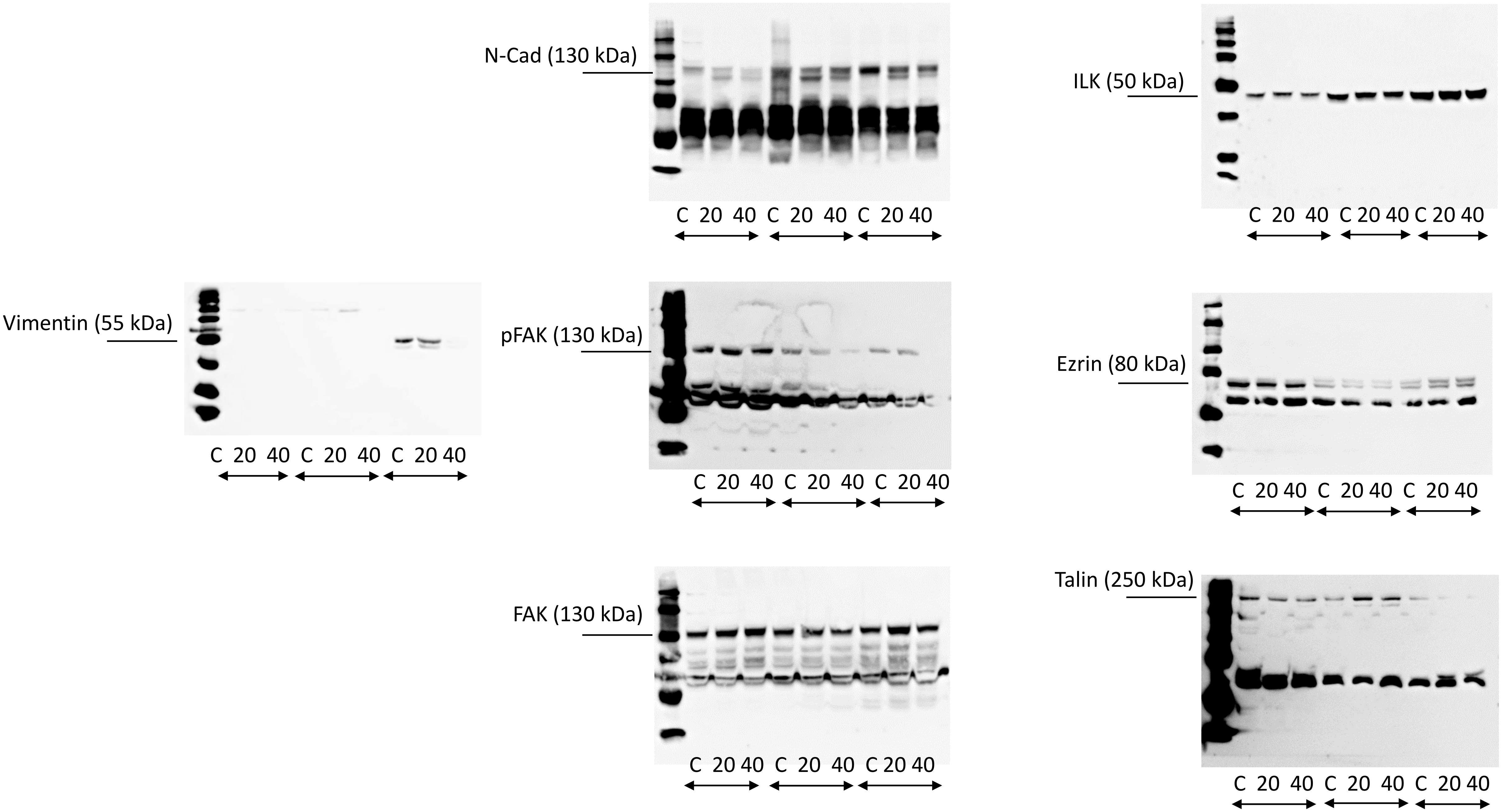

Supplement: Supplementary file 1 [file molecules-31-00555-s001.zip › molecules-4056234-supplementary.pdf]
